# Validation of the Portuguese Variant of the Munich Chronotype Questionnaire (MCTQ^PT^)

**DOI:** 10.3389/fphys.2020.00795

**Published:** 2020-07-14

**Authors:** Cátia Reis, Sara Gamboa Madeira, Luísa V. Lopes, Teresa Paiva, Till Roenneberg

**Affiliations:** ^1^Centro de Medicina de Sono (CENC), Lisbon, Portugal; ^2^Instituto de Saúde Ambiental (ISAMB), Faculdade de Medicina, Universidade de Lisboa, Lisbon, Portugal; ^3^Instituto de Medicina Molecular João Lobo Antunes, Faculdade de Medicina, Universidade de Lisboa, Lisbon, Portugal; ^4^Unidade de Saúde Familiar Mactamã, Administração Regional de Saúde de Lisboa e Vale do Tejo (ARSLVT), Lisbon, Portugal; ^5^Comprehensive Health Research Center (CHRC), Nova Medical School, Faculdade de Ciências Médicas, Lisbon, Portugal; ^6^Institute for Medical Psychology, Medical Faculty, Ludwig-Maximilians University (LMU), Munich, Germany

**Keywords:** phase of entrainment, MCTQ, actimetry, chronotype, validation

## Abstract

**Introduction:**

Differences in the manner circadian clocks entrain to the 24-h day are expressions of different chronotypes that can range from extreme early to extreme late, from proverbial larks to owls. The Morningness Eveningness Questionnaire (MEQ) was one of the first to assess daily preference based on subjective self-assessment – a psychological construct. The later developed Munich Chronotype Questionnaire (MCTQ) uses instead the actual sleep timing to assess chronotype. It calculates the mid-sleep point, halfway between onset and offset on work-free days (MSF), which is then corrected for potential oversleep on free days compensating for sleep debt accumulated over the workweek (MSF_sc_). MSF_sc_ is expressed in local time and is thought to be a proxy for “phase of entrainment” of the circadian clock. The MCTQ-derived chronotype is therefore a biological construct. In the present report, we validate the Portuguese variant (MCTQ^PT^) of the MCTQ. Portugal is of particular interest, since it is thought to consist of especially late chronotypes.

**Methods:**

We have used three methods to assess the timing of daily behavior, namely, the *chronotype* (MCTQ), the *daily preference* (rMEQ), and a *simple self-assessment* (time-of-day type). A total of 80 healthy adults living in Portugal, with age and sex distributed according to the Portuguese population, were recruited. We analyzed 4 weeks of continuous records of actimetry data to validate the MCTQ^PT^ and used the rMEQ to compare between a *biological chronotype* (sleep timing) and a *psychological chronotype* (daily preference). MCTQ variables were analyzed by descriptive statistics; correspondence between measurements was done by Spearman correlations or cross-tabulation; in a subset of 41 individuals, test–retest reliability was assessed.

**Results:**

MCTQ-derived variables (MSF, MSW, MSF_sc_) correlated highly with their counterparts calculated from actimetry (MSW: rho = 0.697; MSF: rho = 0.747; MSF_sc_: rho = 0.646; all *p* < 0.001). The MCTQ assessment of the chronotype showed good test–retest reliability (rho = 0.905; *p* < 0.001). The rMEQ score correlates with MSF_sc_ (rho = −0.695; *p* < 0.001), and the agreement for the self-assessment with the MSF_sc_ was fair (*kw* = 0.386; *p* < 0.001).

**Conclusion:**

The Portuguese variant of the MCTQ revealed to be a reliable questionnaire to assess the chronotype for the Portuguese adult population, as previously reported for other countries.

## Introduction

The 24 h light–dark cycle is a fundamental characteristic of the planet Earth, and as so, it influences the behavior, metabolism, and physiology of species from all phyla, from unicellular organisms (Mergenhagen, 1980) to humans (Logan and McClung, 2019). These rhythms are regulated by an endogenous circadian clock that synchronizes (entrains) to environmental signals (*zeitgebers*) (Aschoff and Pohl, 1978), of which the light–dark cycle is the most relevant (Czeisler et al., 1981; Roenneberg et al., 2007b). Internal clocks, which are referred to as circadian rhythms (Andreani et al., 2015), allow one to anticipate changes in the environment. Circadian rhythms are endogenously generated and persist under constant conditions (Aschoff et al., 1971). Differences in how the human circadian clock entrains to the 24-h day – earlier or later (e.g., in reference to dawn) – are expressions of different chronotypes that range from extreme early to extreme late (Fischer et al., 2017). The concept of a chronotype is becoming increasingly important in epidemiological research (Levandovski et al., 2013), and there are several methods of its assessment (Adan et al., 2012; Putilov, 2017). This is exemplified by the many different terminologies that supposedly refer to the same concept: “circadian typology,” “circadian phenotype,” “daily or diurnal preference,” “morningness–eveningness preference,” or “phase of entrainment.” Despite the many names and concepts, one can identify two major approaches of defining a chronotype.

The individual daily behavior can be considered a personality trait, where individual preference is assessed by ordinal values. This is the approach used in the Morningness–Eveningness Questionnaire (MEQ), the first validated instrument for chronotyping (Horne and Ostberg, 1976). The MEQ is the most commonly used questionnaire, and it is considered the gold standard measure of morningness (Levandovski et al., 2013). Its original version contains 19 questions; a shorter version (rMEQ) of 5 items is becoming increasingly popular (Adan and Almirall, 1991). The MEQ generates a score in which a higher value indicates morningness and a lower value indicates eveningness. A categorization into morning (M-type), neither (N-type), or evening (E-Type) people has been proposed based on cutoff values (Adan and Almirall, 1991; Loureiro and Garcia-Marques, 2015). Nonetheless, the use of pre-established cutoff values is problematic when comparing different populations (Kuhnle, 2006; Randler and Frech, 2006; Levandovski et al., 2013; Roenneberg, 2015; Roenneberg et al., 2015; Fischer et al., 2017).

However, a chronotype has a genetic basis and is influenced by not only age and gender (Roenneberg et al., 2004; Fischer et al., 2017) but also geographic location and light exposure (*zeitgeber* strength) that plays a role in the entrainment process (Roenneberg et al., 2003, 2004; Miguel et al., 2014; Fischer et al., 2017). A chronotype is a product of the circadian clock synchronizing to the 24 h light–dark cycle of our rotating globe. Traditionally, people were exposed to very bright light during the day and close to complete darkness during the night. Over the past decades, however, people are less exposed to natural light on one hand and, on the other hand, illuminate their nights artificially, thereby weakening the *zeitgeber* strength their circadian clocks are exposed to. This leads to free-running conditions [i.e., running according to the individuals’ endogenous circadian period (τ)] (Granada et al., 2013). As a consequence, extreme early chronotypes have become even earlier, and all other chronotypes have become later, widening the chronotype distribution and increasing the difference between the extremes (Roenneberg and Merrow, 2016). Assessing individual internal time (chronotype) as a biological construct is important, since practically all functions in our body are organized by the circadian clock (Roenneberg et al., 2019). Measuring the phase of the circadian clock is difficult, but it can be estimated by measuring the timing of its outputs as biomarkers for the circadian phase (Roenneberg et al., 2019). Such biomarkers are, for example, the nadir of core body temperature or the onset of the melatonin rise measured in dim light (DLMO). DLMO is regarded as the gold standard (Benloucif et al., 2008; Roenneberg et al., 2019) for assessing the circadian phase, but its measurements are cumbersome, costly, and – if not saliva or urine but blood sample is used – invasive; questionnaires that query the daily timing of sleep–wake behavior are therefore more practical, especially in large studies (Roenneberg, 2015).

The Munich ChronoType Questionnaire (MCTQ) was developed as a practical proxy for circadian “phase of entrainment” (Roenneberg et al., 2003), a quantifiable biological phenotype, a state, rather than a psychological trait (Roenneberg et al., 2019). It consists of simple questions asking people to describe their sleep behavior step by step from going to bed to getting up. Notably, these questions are asked separately for workdays (W) and for work-free days (F). This separation is important for the computation of the chronotype. The resulting key MCTQ variables are mid-sleep (the mid-point between sleep onset and offset), sleep duration, and the difference between mid-point of sleep on work- and free days, called social jetlag (SJL) (Roenneberg et al., 2019). Chronotyping is based on the Mid-Sleep time on Free days (MSF), which is corrected for potential compensatory sleep (resulting from sleep deprivation during the workweek; homeostatic component; MSF_sc_). Using behavior on work-free days tries to minimize social influences on sleep timing focusing on the biological circadian influences, rather than on social schedules, like work or school times (Roenneberg et al., 2007a; Roenneberg, 2015). An MCTQ-derived chronotype is expressed in local time (and not a score, as produced by the MEQ). All MCTQ-derived times and durations are continuous variables with population-specific distributions (Roenneberg, 2015).

The MCTQ has been applied in many different countries: Japan (Kitamura et al., 2014); Korea (Suh et al., 2018); Brazil (Miguel et al., 2014; Pilz et al., 2018b); and several European countries: Poland (Jankowski, 2015), Germany (Roenneberg et al., 2003; Kuhnle, 2006), Italy (Ghotbi et al., 2019), and Netherlands (Zavada et al., 2005). The MCTQ has been compared with the MEQ (Zavada et al., 2005; Kitamura et al., 2014; Miguel et al., 2014; Suh et al., 2018) and has been validated against DLMO (Kitamura et al., 2014; Kantermann et al., 2015; Facer-Childs et al., 2019), cortisol (Facer-Childs et al., 2019), and actimetry, for extreme chronotypes (Facer-Childs et al., 2019), for young adults (Santisteban et al., 2018), for communities with different levels of urbanization (Pilz et al., 2018b), and for shift workers (Juda et al., 2013b). Recently, an ultrashort version (μMCTQ) has been created and validated against DLMO and actimetry (Ghotbi et al., 2019).

Here, we introduce and validate the European Portuguese variant (MCTQ^PT^) of the MCTQ. We used actimetry to validate the MCTQ and the rMEQ to compare between a *psychological chronotype* (daily preference) and a *biological chronotype* (sleep timing). Besides providing the Portuguese version of the questionnaire, its application in Portugal is of particular interest due to the specially high prevalence of late chronotypes (Reis and Paiva, 2019).

## Materials and Methods

### Procedures

The original English variant of the questionnaire was translated by two individuals proficient in both English and Portuguese. A consensus version was obtained between the translators and the investigators, which was subsequently back-translated to English by a third translator of equal qualification, essentially producing the same questions as the original (back-translation was approved by the original developers of the MCTQ). We tested comprehensibility, semantic validity, and cultural adequacy of the Portuguese MCTQ variant (MCTQ^PT^) in a preliminary survey, which did not lead to additional text changes. Data were assessed in the context of other experiments. On average, participants took 7 min to complete the MCTQ^PT^.

Our study included sociodemographic questions (age and gender), the MCTQ^PT^, the rMEQ, wrist actimetry (24 h/day for 4 consecutive weeks), and keeping a sleep-log during the 4-week period. In this paper, we apply three methods of assessing the timing of daily behavior: *chronotype* (MCTQ), *daily preference* (rMEQ), and *simple self-assessment* (time-of-day type). With these, we could compare biological and psychological assessments of the chronotype.

For validation purposes, we compared the MCTQ^PT^ results of 80 healthy individuals to the objective sleep–wake assessment of actimetry. Test–retest reliability of the MCTQ^PT^ was also assessed in a subset of 41 participants.

### Participants

A sample of 80 Portuguese-speaking volunteers (age ≥18 and ≤65) living in Portugal was recruited between March 2017 and March 2018. Since the MCTQ needs sleep information for both work- and work-free days, we focused on working adults. Sex and age distribution mirrored the national Portuguese distribution (INE, 2011). Exclusion criteria were pregnancy, raising children under 2 years, as well as working in shifts and having traveled across time zones in the previous 3 months. Data from 80 healthy adults (i.e., with no reported health complaints nor medication) were used for the validation process (42 women and 38 men, with an average age of 38.94 ± 14.90 years). Almost a quarter of the participants (18, 22.5%) used an alarm clock on free days, so that their MSF_sc_ could not be calculated, reducing the sample for MSF_sc_ to 62 (30 men and 32 women, with an average age of 40.40 ± 14.89 years).

All participants gave their written informed consent, and the Lisbon Medical School Ethics Committee approved the study design.

### Subjective Measurements

#### Munich Chronotype Questionnaire (MCTQ)

The MCTQ queries sleep times, and its chronotype is considered a quantifiable circadian trait based on MSF_sc_ (see the Introduction). Note that a chronotype is expressed in local time and can only be calculated if participants report not using alarm clocks on work-free days. Since MSF_sc_ is a continuous variable, a quantile approach was used (Fischer et al., 2017). For the list of variables (see Supplementary Table S1).

#### Reduced Morningness–Eveningness Questionnaire (rMEQ)

The rMEQ is a short version of the MEQ (Adan and Almirall, 1991) that has been validated for the Portuguese population (Loureiro and Garcia-Marques, 2015). The rMEQ results in a total score ranging from 4 to 25 that can be categorized to define different daily preferences: 4–11 for evening-type (E-type), 12–17 for neither or neutral-type (N-type), and 18–25 for morning-type (M-type).

Note that the two instruments have an inverse relationship: an individual with a morning preference leading to a high rMEQ score (i.e., >18) has an early (“low”) mid-sleep time on free days in the MCTQ (i.e., an MSF_sc_ at 2:30).

#### Self-Assessment (Time-of-Day Type)

The time-of-day-type term was already used for a seven-category self-assessment of the chronotype (Roenneberg et al., 2007a). Here, we used four self-report categories in the context of the last rMEQ question: “Which one of these types do you consider yourself to be?” “Definitely a morning-type,” “Rather more a morning-type than an evening-type,” “Rather more an evening-type than a morning-type,” or “Definitely an evening-type” (Jankowski, 2013; Loureiro and Garcia-Marques, 2015).

### Objective Measurements

Locomotor activity was recorded for 4 weeks with wrist-worn devices (Condor Instruments^TM^). Participants could follow their usual routines, in their home and work environment. Activity was sampled every second and stored in 1-min bins; for data analyses in the ChronoSapiens software (vs 11.x; ^©^ Chronsulting UG), data were binned further in 10-min bins. Times of not wearing the actimeter were defined as stretches of at least 10 consecutive bins (100 min) without activity. These “missing data” were excluded from the analysis. We also excluded entire days including ≥4 h of missing data. The shortest time series after data cleaning was 11 days, which contained at least 2 weekends (4 work-free days); 76 participants (97.5%) completed the 4 weeks of actimetry. Information about work- and free days was retrieved from the sleep logs. The actimetry from two individuals had to be discarded for technical reasons. Phase assessments of the activity recordings and derived sleep variables (sleep onset, sleep end, mid-sleep, and sleep duration) were calculated with published algorithms (Roenneberg et al., 2015) using the ChronoSapiens program (^©^Chronsulting UG). Based on these derived sleep variables, MCTQ-derived variables were calculated (e.g., MSF, MSW, MSF_sc_, SJL). We also used the acrophase (center of gravity, φ_max_) of a 24-h cosine fit (Young, 1977) to the daily actimetry profiles as an objective proxy for the chronotype (Roenneberg et al., 2015). To make this phase more compatible with mid-sleep times, we used the bathyphase (acrophase ± 12 h; φ_min_). For the analysis, objective phase markers were separated into workdays and free days. Note that all phases were centered around midnight (i.e., 22:30 = −1.5).

### Statistical Analysis

The MCTQ variables were analyzed using descriptive statistics [mean ± standard deviation (SD) or median with interquartile range (IQR)]. Normality of MCTQ variables, rMEQ scores, and actimetry data was analyzed by the Shapiro-Wilk test. The MCTQ variable comparisons for work- and work-free days were assessed with the paired samples *t*-test. Effects of seasons and sex for quantitative variables were assessed with the ANCOVA test, adjusting for age.

The concordance between the MCTQ and actimetry, the test–retest of the MCTQ, as well as the association between the MCTQ with the rMEQ and age were assessed by Pearson (r) or Spearman (rho) correlations.

In order to assess the discrepancies between actimetry and MCTQ variables, the difference (Δ) for the mid-point of sleep, sleep onset (SO), and sleep end (SE) for work- and work-free days was calculated. The sum of squared differences (SSD) was calculated for the respective differences (Δ). A higher SSD represents a higher discrepancy between actimetry and the MCTQ. Time-of-day-type self-assessment and MSF_sc_ were compared by a cross-tabulation using the respective quartiles of the MSF_sc_ distribution and the four self-report categories. Weighted kappa (kw; linear weights for two ordinal variables; Cicchetti and Allison, 1971) was computed to assess the agreement between the respective quartiles and four self-reported categories (values <0 represent no agreement, and 1, a perfect agreement) using the R package “vcd” (Meyer et al., 2020). In all tests, the significance level was set at *p* ≤ 0.05. All other statistical analyses were performed using SPSS 25 and were graphed with Prism 8 for Macintosh.

## Results

**FIGURE 1:**
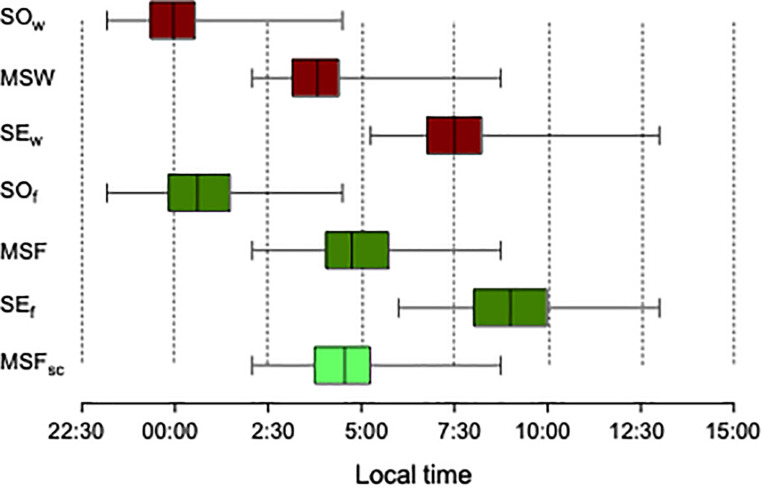
Timing of sleep–wake behavior on workdays (red) and work-free days (green) derived from the MCTQ. The bottom brighter green box represents MSF_sc_. Sample size *n* = 80, except for MSF_sc_ with *n* = 62. SO_w_, Sleep onset on workdays; MSW, Mid-sleep point on workdays; SE_w_, Sleep End on workdays; SO_f_, Sleep onset on free days; MSF, Mid-sleep point on free days; SE_f_, Sleep end on free days; MSF_sc_, Sleep-corrected mid-sleep on free days.

We used the MCTQ^PT^ to assess the sleep–wake behavior on both workdays and free days (Figure 1). Compared to workdays, both free days sleep onset (SO) and sleep end (SE) were delayed by 42 ± 45 min (*t*_79_ = 8.41; *p* < 0.001) and 1 h 24 ± 1 h 19 min (*t*_79_ = 9.46; *p* < 0.001), respectively. Free days sleep duration was on average 42 ± 66 min longer [(*t*_79_ = 5.66; *p* < 0.001; median (IQR): 8.46 (1.54)] than on workdays [median (IQR): 7.67 (1.41)].

**FIGURE 2:**
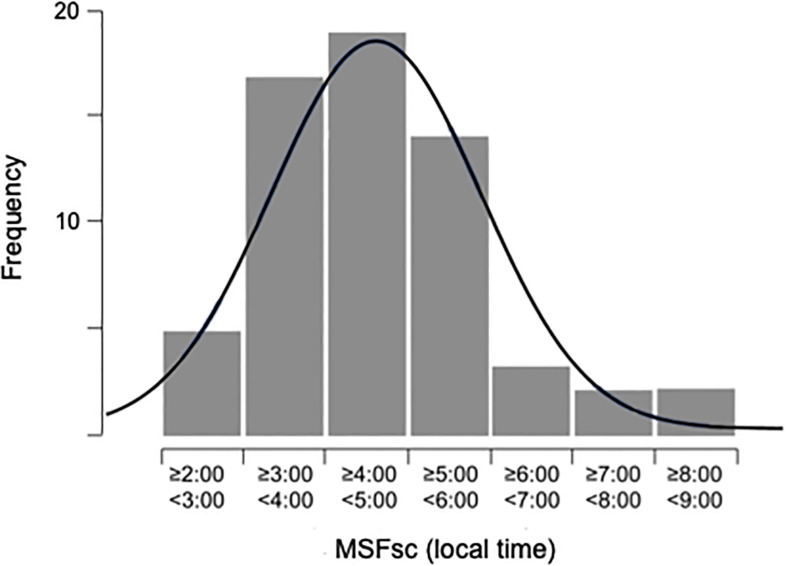
Distribution of chronotype (MSF_sc_; sleep-corrected mid-sleep on free days; local time). Sample size = 62, average age 40.45 ± 14.89 years, ranging from 18 to 65 years.

The chronotype (MSF_sc_) was not normally distributed [median (IQR): 4.55 (1.53) Figure 2]. The respective quartile distribution of the chronotype is shown in Table 1. The chronotype was negatively correlated with age (*r* = −0.455; *p* ≤ 0.001). In the assessment of the effect of sex on the chronotype, women were on average 34 min earlier than men. However, this association was not significant when adjusted for age [*F*(1, 59) = 3.196; *p* = 0.079]. Similarly, mean MSF_sc_ did not differ between seasons [*F*(3, 57) = 0.979; *p* = 0.409] when adjusted for age.

**TABLE 1:** Concordance rate between self-assessment categories and MSF_sc_ quartiles distribution in local time (24 h scale).

		MSF_sc_ quartiles distribution				

Self-assessment	N	2.07–3.72	3.73–4.55	4.56–5.26	5.27–8.75	Concordance rate (%)
“Definitely a morning-type”	5	5	0	0	0	100
“Rather more a morning-type than an evening-type”	36	9	13	10	4	36
“Rather more an evening-type than a morning-type”	18	1	3	6	8	33
“Definitely an evening-type”	3	0	0	0	3	100

**Weighted kappa coefficient (kw = 0.386, 95% CI: 0.239–0.533; p < 0.001).**

Social jetlag (SJL), a measure of circadian misalignment given by the difference between the mid-point of sleep on free days and workdays (SJL = MSF – MSW), was not normally distributed [median (IQR): 0.90 (1.10)].

The distribution of the mid-sleep point on workdays was also not normal [MSW: median (IQR): 3.82 (1.28)], while the mid-sleep point on free days (MSF) was normally distributed (mean: 4.92 ± 1.37).

### Test–Retest Reliability

A subset of 41 participants (16 men; average age of 44.12 ± 14.54 years, range: 18–64) was selected due to their stable lifestyle between subsequent collections (e.g., no travels or changes in work schedule, no vacations, DST change). These participants were asked to complete the MCTQ^PT^ a second time, 2–6 weeks after the initial completion. The MCTQ variables correlated highly between baseline and follow-up [MSF (41) rho = 0.834, MSW (41) rho = 0.831, MSF_sc_ (30) rho = 0.905; all *p* < 0.001].

### Validity Between MCTQ and Actimetry

MCTQ-derived variables were measured by actimetry. Actimetry-derived MSW times were normally distributed (mean: 4.20 ± 1.07) as were those of MSF (mean: 5.10 ± 1.32) and SJL (mean: 0.93 ± 0.91). The distribution of actimetry-derived MSF_sc_ times were, however, skewed [median (IQR): 4.59 (1.53)]. The corresponding variables from the MCTQ and actimetry (MSW, MSF, MSF_sc_, and SJL) correlated highly (MSW: rho = 0.697; MSF: rho = 0.747; MSF_sc_: rho = 0.646; SJL: rho = 0.452; all *p* < 0.001). The lower correlation values were found for sleep duration (SD) for both work- and work-free days (SD_w_: rho = 0.370; SD_f_: rho = 0.343; both *p* < 0.001). For all correlation values for the MCTQ and actimetry (see Supplementary Table S2).

The respective SSD (sum of square differences) between the questionnaire and actimetry for sleep onset (SO), mid-sleep point (MS), and sleep end times (SE) for work- (w) and work-free days (f) were: ΔSO_w_ = 71.67; ΔMSW = 61.53; ΔSE_w_ = 59.62; ΔSO_f_ = 77.86; ΔMSF = 58.04; ΔSE_f_ = 89.76.

**FIGURE 3:**
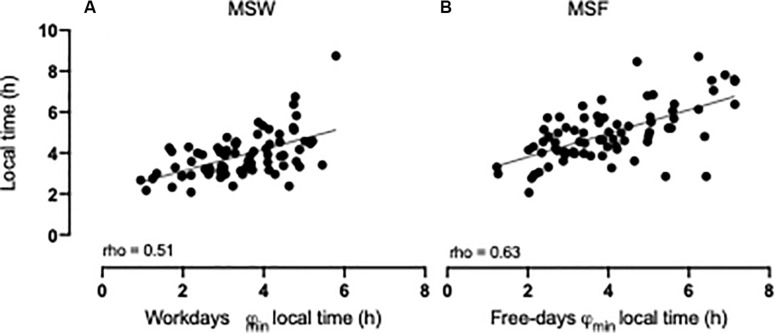
Spearman’s rho correlations for: **(A)** workdays φ_min_ and mid-sleep on workdays (MSW) rho = 0.51; *p* < 0.001; **(B)** free days φ_min_ and mid-sleep on free days (MSF) rho = 0.63; *p* < 0.001. Sample size *n* = 78. For further correlation data (see Supplementary Table S2).

The phase of minimal activity (φ_min_) was normally distributed both on work- (mean: 3.73 ± 1.03) and free days (mean: 4.71 ± 1.30). Both workday and free day φ_min_ advanced with age (*r* = −0.556 and *r* = −0.603, respectively; both *p* < 0.001), corroborating previous findings (Roenneberg et al., 2004; Fischer et al., 2017), but did not show sex differences while adjusting for age [*F*(1, 75) = 0.120; *p* = 0.730 and *F*(1, 74) = 3.648; *p* = 0.060, respectively]. Both MCTQ-derived MSW and MSF correlated highly with their respective φ_min_ values (MSW: rho = 0.507; MSF: rho = 0.629; both *p* < 0.001; Figure 3).

### MCTQ Comparison With rMEQ and Simple Self-Assessment (Time-of-Day-Type)

**FIGURE 4:**
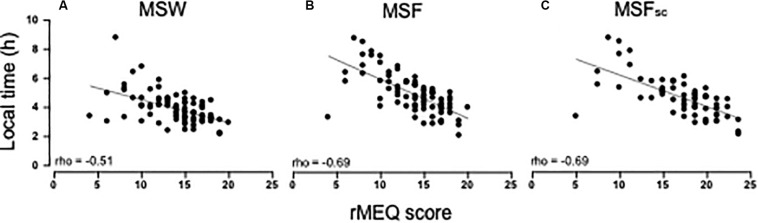
Spearman’s rho correlations between the rMEQ score and MCTQ variables: **(A)** MSW (mid-sleep on workdays; rho = −0.51; *p* < 0.001); **(B)** MSF (mid-sleep on free days; rho = −0.69; *p* < 0.001); and **(C)** MSF_sc_ (sleep-corrected mid-sleep on free days; rho = −0.69; *p* < 0.001). Sample size *n* = 80 except for MSF_sc_ (*n* = 62).

The distribution of the rMEQ scores was skewed [median (IQR): 14.0 (4.0)]. rMEQ scores correlated positively with age (rho = 0.311; *p* = 0.001) and showed sex differences (*U* = 576.5; *p* = 0.032), with women scoring higher scores (morning preferences). However, when adjusting for age, this association was not significant [*F*(1, 77) = 2.800; *p* = 0.098]. rMEQ scores correlate highly with the MCTQ-derived variables (MSW: rho = −0.505; MSF: rho = −0.690; MSF_sc_: rho = −0.695; all *p* < 0.001; Figure 4).

**FIGURE 5:**
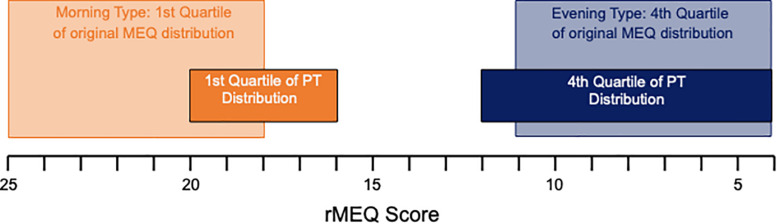
The rMEQ distribution score ranging from 4 to 25 (scores are plotted analogous to Figure 1 with earlier chronotypes – higher scores – on the left and later chronotype – lower scores – on the right). Dim boxes show the original quartiles and dark boxes those of our Portuguese sample.

The original reduced Morningness–Eveningness cutoffs were calculated using a Spanish population (Adan and Almirall, 1991) with M-type >18 and E-type <11. The rMEQ quartiles of the Portuguese population used here result in M-type >16 and E-type <12 (Figure 5). According to the original cutoffs for the rMEQ, the great majority of participants were classified as N-type 50 (62.5%), followed by E-type 20 (25.0%) and M-type 10 (12.5%).

According to the self-assessment of the time-of-day type, the majority consider themselves as “rather more a morning-type than an evening-type” (43, 53.8%), followed by “rather more an evening-type than a morning-type” (26, 32.5%), followed by “definitely a morning-type” (8, 10%) and “definitely an evening-type” (3, 3.8%). The agreement for the four categories of self-assessment with the MCTQ-MSF_sc_ quartiles distribution had a fair agreement for the two measures (kw = 0.386, 95% CI: 0.239–0.533; *p* < 0.001) (Table 1).

## Discussion

Our results show that the MCTQ^PT^ is a valid instrument to assess the chronotype (phase of entrainment) in the population of Portugal and that this subjective assessment corresponds well with objective actimetry.

### Validation of the MCTQ^PT^ Against Actimetry

The phase of minimal activity on free days (φ_min_ of one-harmonic cosine fit) is highly correlated with the MCTQ-derived MSF. The corresponding workday correlation was slightly lower but also highly significant. Comparisons between MCTQ results and general daily activity profiles are assumption-free, e.g., no secondary sleep detection is required. However, specific activities (e.g., biking to work or jogging) affect the timing of the cosine fit.

To make more direct comparisons, we assessed objective sleep times within the activity recordings (Roenneberg et al., 2015) and calculated the variables corresponding to those generated by the MCTQ (e.g., MSF and MSW). The direct association between sleep parameters was even stronger than for the global activity fit. Again, free days correlated higher than those for workdays. While mid-sleep times showed moderate to strong correlations, sleep durations showed weaker correlations. A smaller discrepancy (lowest sum of square difference) between the questionnaire and actimetry was found for the mid-sleep point on free days, indicating a higher stability for this phase measure in comparison to sleep onset and sleep end. Mid-point has generally been thought to represent the entrained phase more accurately than sleep onset or offset (Terman et al., 2001), which suggests that mid-points are less susceptible to the homeostatic regulation of sleep. It is unlikely that difficulties in onset and offset detection are responsible for mid-points showing higher correlations than durations, since they would have to affect both onsets and offsets almost randomly to produce this result. It should also be noted that questionnaires – despite being subjective – may reflect general behavior averaged over time, while measurements (e.g., actimetry or melatonin) – despite being objective – always represent an acute state at the time of the measurement.

We did not validate against dim-light melatonin onset (DLMO), the gold standard for the circadian phase; however, in a recent validation study (Ghotbi et al., 2019), MSF_sc_ showed stronger correlations for actimetry than for DLMO.

Strong correlations between actimetry data and MCTQ variables have been previously shown, for example, for MSF (*r* = 0.57, *p* < 0.001) in Portuguese-speaking Brazilian communities with different levels of rural urbanization (Pilz et al., 2018b). A recent validation study of a shorter MCTQ version (μMCTQ; Ghotbi et al., 2019) produced correlation levels similar to the ones found here (e.g., MCTQ-derived MSF_sc_ *r* = 0.52, *p* < 0.01).

The MCTQ^PT^ showed high test–retest reliability similar to and with values within the same range as reported for German and Korean populations (Kuhnle, 2006; Suh et al., 2018). There were, however, important differences: the Koreans had a female-only sample (Suh et al., 2018), and Kuhnle’s sample was restricted to university students and was only assessed for MSF (Kuhnle, 2006).

### Chronotype Characteristics of the Portuguese Population

In our study, the weekly average sleep duration was of 7 h and 53 min (no reports < 5 h), which is in line with a 7- to 9-h recommendation for the considered age group (Hirshkowitz et al., 2015). In a recent Portuguese study with national representativeness (Reis et al., 2018), over 20% subjectively stated short sleep duration (≤ 5 h). However, that study did not separate between work and free days, which is known to bias the subjective assessments toward the more numerous workdays (Roenneberg et al., 2007a; Pilz et al., 2018a) that usually are characterized by shorter sleep (Roenneberg, 2013), as was also the case in the present study.

A chronotype advances with age (Roenneberg et al., 2004, 2007a). The latest chronotypes are found around the age of 20, a tendency also observed in our adult population (*r* = −0.455; *p* < 0.001). These MCTQ results were also supported by the φ_min_, which showed a strong negative correlation with age (workdays: *r* = −0.556, *p* < 0.001; free days: *r* = −0.603, *p* < 0.001).

Men are generally later than women (Roenneberg et al., 2007a), which is also true for our Portuguese sample. However, this sex difference did not reach significance for the MCTQ, as well as for the φ_min_.

### Comparing the Portuguese MCTQ With That of Other Countries

Earlier reports suggested that the Portuguese population is especially late (Reis and Paiva, 2019), which can be scrutinized in the current study.

According to these results, Koreans and Italians are – on average – later chronotypes than Portuguese. However, the ages of the participants in the respective studies bias the average chronotype of Koreans and Italians toward later chronotypes (Table 2).

**TABLE 2:** Country comparison of different studies representing the MCTQ average chronotype.

Country	MSF_sc_	Average age	References
Korea	5.13 ± 1.54	27.09 ± 5.64	Suh et al., 2018
Italy	4.75 ± 1.22	31.30 ± 13.00	Ghotbi et al., 2019
Portugal	4.63 ± 1.39	40.40 ± 14.89	Present study
Germany	4.40 ± 1.44	33.91 ± 12.96	MCTQ database
Japan	4.31 ± 0.07	35.69 ± 11.92	Kitamura et al., 2014

**MSF_*sc*_ and age are given for mean ± standard deviation values; the countries are ordered from late to early.**

Our participants were mainly from urban areas. This precludes direct comparison of our study with a recent Brazilian Portuguese-speaking study using the MCTQ, since that was developed within rural communities with different levels of urbanization (i.e., access to electrical light) (Pilz et al., 2018b). Further studies comparing the chronotype in different countries controlling for age, sex, photoperiod (season × latitude), position in time zone, and other confounders are necessary.

### MCTQ Comparison With rMEQ and Simple Self-Assessment (Time-of-Day-Type)

Although the MCTQ and the rMEQ evaluate different traits of a chronotype, the results for these instruments correlated well, according to what has been shown before (Zavada et al., 2005). MSF_sc_ in our sample correlated slightly higher than MSF contrary to previous publications, where the higher correlation values were found for MSF (Roenneberg et al., 2007a; Kitamura et al., 2014; Suh et al., 2018). Correlations between the rMEQ score and both MSF_sc_ and MSF were in the same range for the different populations (Portugal, present study): MSF_sc_ rho = −0.695; MSF rho = −0.690; Korea (Suh et al., 2018): MSF_sc_ *r* = −0.546; MSF *r* = −0.571; Japan (Kitamura et al., 2014): MSF_sc_ *r* = −0.612; MSF *r* = −0.652.

We also compared the MSF_sc_ with the individual’s self-assessment of time-of-day type (Table 1). There was a fair concordance between each of the four options of self-assessment and the MCTQ quartile distribution according to the published classification (Fleiss and Cohen, 1973). Interestingly, some individuals classified themselves as “rather more an evening type than a morning type” in the self-assessment while they fell in the last quartile of the MSF_sc_ (i.e., late type).

Our Portuguese sample has a later daily preference in comparison to the established cutoff values for the rMEQ (Adan and Almirall, 1991; Loureiro and Garcia-Marques, 2015). Even for a heterogeneous adult sample, scores were, in general, lower (i.e., maximum score 25 vs. 20; Figure 5), highlighting the limitation of pre-established cutoffs; average scores may be population-/sample-specific.

## Conclusion

The Portuguese variant of the MCTQ correlates well with actimetry and has high reliability. If we consider that there are around 290 million Portuguese language speakers around the world, the MCTQ^PT^ is an important instrument and an excellent chronotype assessment method – simple, short, and non-invasive. It is particularly useful to assess the chronotype among different countries, since it allows enhancing the knowledge of human phase of entrainment in real-life settings. In addition, the chronotype impacts both sleep quality (Reis et al., 2020) and sleep duration (Juda et al., 2013a), mediated by SJL. The growing awareness for health consequences associated with circadian misalignment (discrepancies between internal and external time due to modern society lifestyles) requires simple methods to assess circadian timing and quantify the consequent SJL.

## Data Availability Statement

The datasets presented in this article are not readily available because they are still part of an ongoing study. Requests to access the datasets should be directed to the corresponding author.

## Ethics Statement

The studies involving human participants were reviewed and approved by the Lisbon Medical School Ethics Committee. The patients/participants provided their written informed consent to participate in this study.

## Author Contributions

CR, SM, LL, TP, and TR contributed to the design and implementation of the study. CR and SM collected the data, performed the statistical analysis, and wrote the manuscript. CR treated the actimetry data. TR supervised the study. All authors contributed to the article and approved the submitted version.

## Conflict of Interest

The authors declare that the research was conducted in the absence of any commercial or financial relationships that could be construed as a potential conflict of interest.
