# Supplementary material for: Validation of the Portuguese Variant of the Munich Chronotype Questionnaire (MCTQPT)
Source: Front Physiol. 2020 Jul 14;11:795. doi: 10.3389/fphys.2020.00795 (PMC7372122; doi:10.3389/fphys.2020.00795)
Supplement: Supplementary file 1 [file Table_1.DOCX]

**Validation of the Portuguese variant of the Munich Chronotype Questionnaire (MCTQ^PT^)**

Cátia Reis, Sara Madeira, Luísa V. Lopes, Teresa Paiva, Till Roenneberg

**Supplementary material**

Content:

1. Table S1: List of MCTQ variables and MCTQ variable computation. Can also be downloaded from: <https://thewep.org/documentations/mctq/item/mctq-variables>

2. Table S2: Correlation values between MCTQ questionnaire variables and the respective actimetry sleep derived data calculated variables and phase of activity (φ_min_)

Table S1: List of MCTQ variables and MCTQ variable computation.

|  |  |  | **Workdays** | | **Work-free days** | |
| --- | --- | --- | --- | --- | --- | --- |
| **Name** | **Statement** | **Format** | **Abbreviation** | **Computation** | **Abbreviation** | **Computation** |
| **Basic variables** | | | | | | |
| Local time of going to bed | I go to bed at… o’clock. | hh:mm | BT_w_ | - | BT_f_ | - |
| Local time of preparing to sleep | I actually get ready to fall asleep at… o’clock. | hh:mm | SPrep_w_ | - | SPrep_f_ | - |
| Sleep latency | I need…minutes to fall asleep. | mm | SLat_w_ | - | SLat_f_ | - |
| Sleep end | I wake up at… o’clock. | hh:mm | SE_w_ | - | SE_f_ | - |
| Alarm clock use | with an alarm clock/without alarm clock | y/n | Alarm_w_ | - | Alarm_f_ | - |
| Sleep inertia | After… minutes, I get up. | mm | Sl_w_ | - | Sl_f_ | - |
| Number of work-/work-free days | I have a regular work schedule and work… days per week. | n | WD | - | FD | 7-WD |
| Light exposure | On average, I spend the following amount of time outdoors in daylight (without a roof above my head) | hh:mm | LE_w_ | - | LE_f_ | - |
| **Computed variables** | | | | | | |
| Sleep onset | - | hh:mm | SO_w_ | SPrep_w_ + SLat_w_ | SO_f_ | SPrep_f_ + SLat_f_ |
| Local time of getting out of bed | - | hh:mm | GU_w_ | SE_w_ + Sl_w_ | GU_f_ | SE_f_+Sl_f_ |
| Sleep duration | - | hh:mm | SD_w_ | SE_w_ - SO_w_ | DS_f_ | SE_f_ - SO_f_ |
| Total time in bed | - | hh:mm | TBT_w_ | GU_w_ - BT_w_ | TBT_f_ | GU_f_ - BT_f_ |
| Mid-Sleep | - | hh:mm | MSW | SO_w_ + SD_w_/2 | MSF | SO_f_ + SD_f_/2 |
| **Computed variables combining workdays and work-free days** | | | | | | |
| **Name** | | **Format** | **Abbreviation** | | **Computation** | |
| Average weekly sleep duration | | hh:mm | SD_week_ | | (SD_w_ x WD + SD_f_ x FD)7 | |
| Chronotype (only computed if Alarmf = no) | | hh:mm | MSF_sc_ | | If SD_f_ ≤ SD_w_:  MSF  If SD_f_ > SD_w_:  MSF – (SD – SD_week_)/2 | |
| Weekly sleep loss | | hh:mm | Sloss_week_ | | If SD_week_ > SD_w_:  (SD_week_ – SD_w_) x WD | |
| Relative social jetlag | | hh:mm | SJL_rel_ | | MSF – MSW | |
| Absolute social jetlag | | hh:mm | SJL | | \| MSF – MSW \| | |
| Average weekly light exposure | | hh:mm | LEweek | | (LE_w_ x WD + LE_f_ x FD)/7 | |

Table S2: Correlation values between MCTQ questionnaire variables and the respective actimetry sleep derived data calculated variables and phase of activity (φ_min_)

All results are given for the Spearman’s correlation test, besides having some variables assuming normal distribution (see figure 3) in order to standardize results since most variables assumed a non-parametric distribution. *p<0.05; **p<0.01; the bold values are the correspondent for both measurements (MCTQ questionnaire and actimetry derived data). n.a. – non applicable.
